# Sustainable Development Goals (SDG)

**DOI:** 10.1128/msystems.01140-23

**Published:** 2024-01-08

**Authors:** Jack Gilbert

**Affiliations:** 1 University of California, San Diego, La Jolla, California, USA

## EDITORIAL

There is no denying the gravity and importance of the United Nations Sustainable Development Goals (UN SDGs)—a clarion call for worldwide collaboration aimed at addressing our planet's most pressing challenges by 2030. The 17 goals, ranging from eradicating poverty to establishing responsible consumption and production patterns, represent a unified, ambitious blueprint for a better, more sustainable future.

Despite their obvious importance, microbial systems have not been a traditional focal point when discussing the SDGs. However, since 2019, a series of groundbreaking articles published in *mSystems* have begun to underscore the profound intersections between the microbial world and these global sustainability targets. We are excited to introduce a special collection that curates these articles, offering readers an in-depth look into how microbial science contributes knowledge and understanding to the realization of several SDGs.

Microorganisms play an indelible role in our environment, health, industries, and daily life. The myriad applications of microbial systems—from waste recycling and clean energy production to agriculture and disease control—are testament to their versatility and importance. Yet, their potential in aiding our sustainability efforts has often been overlooked or underemphasized. By collating these articles under a dedicated collection, we aim to bring to the fore the numerous ways in which microbial systems can propel our knowledge base to facilitate achieving the SDGs.

This collection seeks to educate and inform researchers, policymakers, and the public of the latest advancements in microbial systems pertinent to the SDGs. We also hope that this collection, which will be routinely updated, will inspire collaborations by identifying scientific neighborhoods around the UN SDGs to catalyze interdisciplinary dialogues and partnerships. Finally, we hope to drive further research by explicitly highlighting current successes and applications.

Several articles within this collection unveil novel insights. For instance, there’s research elucidating the role of microbiomes in food as medicine, enhancing agricultural productivity and reducing greenhouse gas emissions (supporting Goal 2: Zero Hunger and Goal 13: Combat Climate Change) and studies on microbial innovations that help in providing clean water (resonating with Goal 6: Clean Water and Sanitation). Additionally, we have studies that focus on biodiversity and sustainability in the water and on land, addressing Goal 14: Life under Water and Goal 15: Life on Land. Many of the papers address multiple SDGs, which is testament to their multidisciplinary focus.

The creation of this special collection is not just an academic exercise but a commitment from *mSystems* to improving global sustainability. We believe that in understanding and leveraging microbial systems biology, we move a step closer to fulfilling the promise of the UN SDGs. As you peruse this collection, we invite you to appreciate the intricacies and enormities of the microbial world and its pivotal role in our shared sustainable future.

